# Study of Kroenig’s Isthmus in Pulmonary Tuberculosis

**Published:** 1916-03

**Authors:** 


**Study of Kroenig's Isthmus in Pulmonary Tuberculosis.** W. C. Klotz, Los Angeles, Boston M. & S. Jour., Dec. 9, 1915. Allusion is made to Fetterolf and Norris's investigations of frozen cadavers, showing that the right apex is regularly less developed than the left, without regard to right-handedness, and undoubtedly due to encroachment of the large blood vessels of the neck and the position of the trachea. Klotz first determines the area of transmitted pulmonary resonance, as described by Kroenig, marking with dermograph from the clavicle in front to the spine of the scapula. These are transcribed to case charts and measurements made. If the pulmonary resonance does not reach the upper margin of the trapezius, Kronig's isthmus is designated as zero. 2.5 and 5 c. m., 4 and 7 c. m., 0 and 5 c. m. are mentioned as actual findings. He tabulates his findings as follows: Right sided lesions, 44, right isthmus narrower in 43, left side narrower in 1. Left sided lesions, 18, right isthmus narrower in 14, left in 4. Bilateral lesions 67, right isthmus narrower in 66, left in 1. In 21 normal controls, the right isthmus was narrower in 18, the left in 1, both the same in 2. (Note: As we understand the matter, the word isthmus is not appropriate. The normal differences between the two pulmonary apexes have been well understood for many years, both anatomically and as regards physical signs and the discounting of this difference in pulmonary tuberculosis and other lesions has also been long taught. Even the mapping out of the apexes, by palpatory thrill, breath and voice signs, ordinary and auscultatory percussion has been practiced by many physicians.—Ed.).

